# Revisiting an *Aspergillus flavus* Strain Isolated from an Egyptian Sugarcane Field in 1930

**DOI:** 10.3390/microorganisms8111633

**Published:** 2020-10-22

**Authors:** Mohamed F. Abdallah, Kris Audenaert, Sarah De Saeger, Jos Houbraken

**Affiliations:** 1 Centre of Excellence in Mycotoxicology and Public Health, Department of Bioanalysis, Faculty of Pharmaceutical Sciences, Ghent University, B-9000 Ghent, Belgium; sarah.desaeger@ugent.be; 2 Laboratory of Applied Mycology and Phenomics, Department of Plants and Crops, Faculty of Bioscience Engineering, Ghent University, B-9000 Ghent, Belgium; kris.audenaert@ugent.be; 3 Department of Forensic Medicine and Toxicology, Faculty of Veterinary Medicine, Assiut University, Assiut 71515, Egypt; 4 Westerdijk Fungal Biodiversity Institute, Uppsalalaan 8, NL-3584 CT Utrecht, The Netherlands; j.houbraken@wi.knaw.nl

**Keywords:** sugarcane, *A. novoparasiticus*, aflatoxins, *Pseudococcus sacchari*, polyphasic approach, leporins, climate change

## Abstract

The aflatoxin type B and G producer *Aspergillus novoparasiticus* was described in 2012 and was firstly reported from sputum, hospital air (Brazil), and soil (Colombia). Later, several survey studies reported the occurrence of this species in different foods and other agricultural commodities from several countries worldwide. This short communication reports on an old fungal strain (CBS 108.30), isolated from *Pseudococcus sacchari* (grey sugarcane mealybug) from an Egyptian sugarcane field in (or before) 1930. This strain was initially identified as *Aspergillus flavus*; however, using the latest taxonomy schemes, the strain is, in fact, *A. novoparasiticus*. These data and previous reports indicate that *A. novoparasiticus* is strongly associated with sugarcane, and pre-harvest biocontrol approaches with non-toxigenic *A. novoparasiticus* strains are likely to be more successful than those using non-toxigenic *A. flavus* strains. Further studies on the association between *A. novoparasiticus* and *Pseudococcus sacchari* might shed light on the distribution (and aflatoxin contamination) of this species in sugarcane. Additionally, the interaction between *A. novoparasiticus*, *Pseudococcus sacchari,* and sugarcane crop under different scenarios of climate change will be critical in order to get more insight into the host–pathogen interaction and host resistance and propose appropriate prevention strategies to decrease mycotoxin contamination and crop loss due to *A. novoparasiticus* attack.

## 1. Introduction

Egypt started sugarcane (*Saccharum officinarum L.*) cultivation in 1848 when the first variety of the crop was imported from Jamaica. Since that time, sugarcane is cultivated for three main purposes: human consumption (chewing), sugarcane honey manufacturing in villages for local use, and sugar production. In the years 1922-23, the production of sugar severely dropped due to an extreme attack of the grey sugarcane mealybug *Pseudococcus sacchari* Ckll. Two main reasons for that outbreak were given by the entomologist Mr. Wilfrid J. Hall—(1) bad cultivation procedures and (2) introduction of an external cultivar of low quality (called “105” Java cane), which favored the increase of *Pseudococcus sacchari* infestation in the sugarcane field. Interestingly, an association between the cane, the *Pseudococcus sacchari* insect, and a green fungus was observed. Mr. Hall found that in some cases, at least 50% of the *Pseudococcus sacchari* insects on a cane had been killed by this green fungus, which was growing on and immediately around the insects. The fungus was identified as *Aspergillus flavus*, which was known in several regions of the North Atlantic Ocean and the Caribbean as a biocontrol agent against *Pseudococcus sacchari* [1]. This was before the discovery of aflatoxins (AFs), fungal polyketide hepatocarcinogenic secondary metabolites, at the beginning of the 1960s [2,3,4]. After that, scientists have been conducting extensive research to fully understand AFs toxicity and their interaction with other mycotoxins and other food contaminants inside the animal and human bodies and to develop effective tools to mitigate them either in pre- and/or post-harvest stage(s).

Aflatoxin type B (AFB_1_ and AFB_2_) and/or type G (AFG_1_ and AFG_2_)-producing species are mainly classified in *Aspergillus* section *Flavi* and include *Aspergillus aflatoxiformans*, *Aspergillus arachidicola*, *Aspergillus austwickii*, *Aspergillus cerealis*, *Aspergillus flavus*, *Aspergillus luteovirescens*, *Aspergillus minisclerotigenes*, *Aspergillus mottae*, *Aspergillus nomius*, *Aspergillus novoparasiticus*, *Aspergillus parasiticus*, *Aspergillus pipericola*, *Aspergillus pseudocaelatus*, *Aspergillus pseudonomius*, *Aspergillus pseudotamari*, *Aspergillus sergii*, *Aspergillus togoensis,* and *Aspergillus transmontanensis* [5]. However, many other species related to the genus *Aspergillus* have been misidentified as AFs-producing species [6]. Correct identification of these fungi to species level using morphological characters is not an easy task, even for experienced mycologists, and sometimes can result in misidentification. Food mycologists and taxonomists, therefore, recommend using sequence data for accurate species identification, ideally supplemented with phenotype, physiology, and extrolite data (polyphasic approach) for the accurate description of the isolates [7].

A recent study has shown that *A. novoparasiticus* is the predominant *Aspergillus* section *Flavi* species isolated from sugarcane juice sold in Egypt [8]. An old *Aspergillus* strain, previously identified as *A. flavus*, was isolated from *Pseudococcus sacchari* present in an Egyptian sugarcane field in or before 1930. Therefore, it was our interest to revisit this strain and re-identify it using a polyphasic approach that includes sequence data, morphology, physiology, and the chemical analysis of the secondary metabolites or exo-metabolites produced by the fungus.

## 2. Materials and Methods

The examined fungal strain, CBS 108.30 (= DTO 407-H4), isolated from *Pseudococcus sacchari* on *Saccharum officinarum* in Egypt in or before 1930, was maintained in the CBS culture collection housed at the Westerdijk Fungal Biodiversity Institute, the Netherlands. Other similar strains DTO 099-G4 (ex soil, Tunisia), CBS 126849^T^ (= DTO 223-C3; ex sputum, Brazil), CBS 126850 (= DTO 223-C5; ex-air, Brazil), DTO 421-C2, DTO 421-C3, DTO 421-C4 (all sugarcane juice, Egypt) were also included in the current work as references. The study of the morphology, physiology, and molecular identification was performed, as previously described in the literature [7,9]. In short, a part of the calmodulin (*CaM*) gene was sequenced for molecular identification of the strains at the species level. The morphological intra-species variation was studied by growing the strains for 7-days incubation at 25 °C on the agar media Czapek yeast extract agar (CYA), malt extract agar (MEA), yeast extract sucrose agar (YES), dichloran 18% glycerol agar (DG18), and creatine agar (CREA). Furthermore, the growth rate on CYA at 37 °C and 42 °C was also determined.

For extrolite analysis, all the chemicals and reagents used in the analysis were of analytical grade. Methanol (LC-MS grade), formic acid (99%, LC-MS grade), and acetonitrile (LC-MS grade) were purchased from BioSolve Chimie BV (Valkenswaard, The Netherlands). Ethyl acetate and dichloromethane were purchased from Acros Organics (Geel, Belgium). Sodium hydroxide (max. 0,0002% K) was supplied by Merck (Darmstadt, Germany). Ultrapure H_2_O was obtained from an arium mini from Sartorius AG (Goettingen, Germany). The strain was subcultured on CYA and YES at 25 °C for 7 days, as recommended [7]. After growing, six plugs (three plugs from the center of the colonies and three from the margins) were collected in a glass test tube and subjected to extrolite extraction by adding 10 mL of a mixture of methanol:dichloromethane:ethyl acetate 10:20:30 (*v*/*v*/*v*) containing 1% of formic acid [7]. The mixture was vortexed using a Labinco L46 vortex-mixer (Labinco B.V., Breda, The Netherlands) and shacked for 45 min using an overhead shaker (Edmund Bühler GmbH, Hechingen, Germany). Afterward, 5 mL was transferred into another glass test tube and subjected to evaporation under a gentle stream of nitrogen gas at 40 °C until complete dryness using a turbovap LV (Biotage, Uppsala, Sweden) and re-dissolved with 500 µL of pure methanol. The glass tube was vortexed for 2 min and then sonicated through Branson 3510-DTH Ultrasonic Cleaner (Fisher Scientific, Belgium) for 30 min. After sonication, all the contents were transferred into 0.22 µm centrifugal filter units (Merck Millipore, Tullagreen, Ireland) and centrifuged at 5000 rpm for 5 min (Sigma 3-18K, GmbH, Germany). From the filtered extract, 150 µL was transferred into an injection vial, and the sample was injected into a Synapt G2−Si High Definition instrument, which is a hybrid quadrupole orthogonal acceleration time of flight equipped (QTOF HRMS) with traveling wave ion mobility separation mass spectrometer (Waters Corporation, Milford, MA, USA) for untargeted analysis, according to Klitgaard et al. (2014) with some modifications [10].

All systems were controlled using MassLynx version 4.1 (Waters Corporation, Milford, MA, USA). The instrument was operated in resolution mode (>20k FWHM) and calibrated with sodium formate clusters (prepared according to the manufacture’s procedure for calibrations up to m/z ~1500). Leucine enkephalin, a lyophilized peptide, was also used as reference material for mass correction by generating the reference ion ((M+H)^+^ = 556.2771). The mass spectrometry parameters were as follows: capillary voltage 2.8 kV; sample cone voltage 40 V; source offset 80 °C; source temperature 130 °C; desolvation gas flow 800 L/h at a temperature of 550 °C, and cone gas flow 50 L/h. Nitrogen was used as the desolvation and cone gases at 6.5 bar. Argon was employed as the collision gas at a pressure of 9.28 × 10^−3^ mbar. Data type was a continuum, and data-dependent acquisition (DDA) mode on positive ion polarity using electrospray ionization (ESI^+^) was chosen and adjusted to 5 ions for MS/MS from a single MS survey scan. Collision energy ramp was selected for fragmentation of ions in the trap cell for the low and high mass from 11/13 V (start/end) to 50/120 V (start/end), respectively. The MS survey scanning time was 0.12 s, while the MS/MS scan time was adjusted to 0.1 s. The MS/MS scan was switched off in case the accumulated total ion chromatogram (TIC) threshold reached 7,000,000 or after 0.2 s. The mass spectra were chosen from m/z 50 to 1200 Da. The lock spray properties were set as follows: scan time of 0.05 s, a lock spray frequency of 20 s, and scans to average three with mass window ±0.5 over a 15 min run time. Chromatographic separation was performed using an ACQUITY UPLC system equipped with a flow-through needle autosampler (Waters Corporation, Milford, MA, USA). A sample volume of 5 µL was injected into an HSS T3 column (1.8 µm, 2.1 × 100 mm, Waters Corporation, Milford, MA, USA) held at 40 °C, while sample temperatures were kept at 7 °C. A linear gradient elution program with solvent A (ultra−pure water, 20 mmol L^−1^ formic acid) and solvent B (acetonitrile, 20 mmol L^−1^ formic acid) was applied with a flow rate of 0.35 mL/min as follows: 90% A and 10% B for 0.5 min, followed by an increase to 100% B from 0.5 to 10.0 min, 100% B maintained from 10.0 to 13.0 min, direct back to 90% A from 13.0 to 13.1 min, and maintaining starting conditions from 13.1 to 15 min [7,10].

Identification of secondary metabolites produced by the fungal strain was done, putatively by dereplication and relying on the accurate mass QTOF HRMS data, using an in-house database (UNIFI software, Waters, UK). Where possible, reference analytical standards were injected, and mycotoxins and other secondary metabolites were identified by comparing retention time and HRMS and HRMS/MS spectra with the corresponding authentic analytical standards.

## 3. Results and Discussion

**Figure 1:**
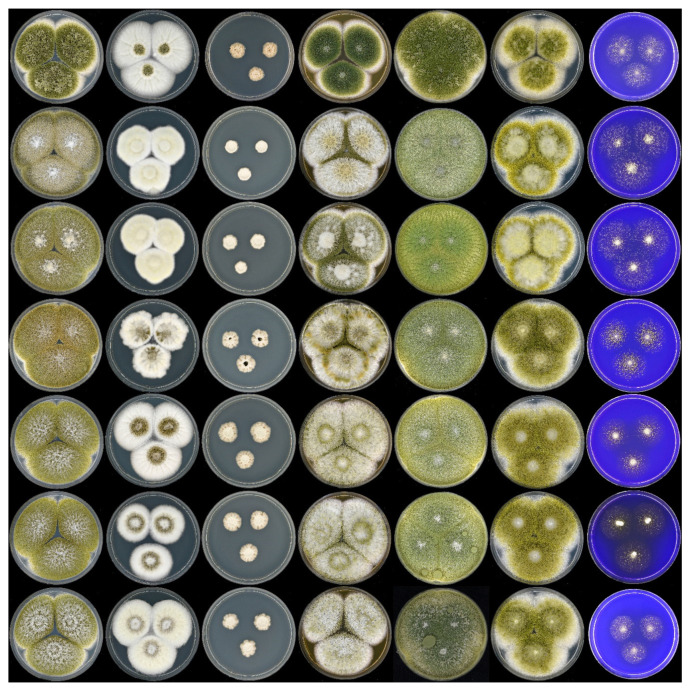
Left to right: 7 d old colonies on CYA, CYA 37°C, CYA 42°C, MEA, YES, DG18, and CREA; top to bottom (all *A. novoparasiticus*): DTO 099-G4, CBS 126849^T^ (= DTO 223-C3), CBS 126850 (= DTO 223-C5), CBS 108.30 (= DTO 407-H4), DTO 421-C2, DTO 421-C3, DTO 421-C4. CYA, Czapek yeast extract agar; MEA, malt extract agar; YES, yeast extract sucrose agar; DG18, dichloran 18 % glycerol agar; CREA, creatine agar.

**Figure 2:**
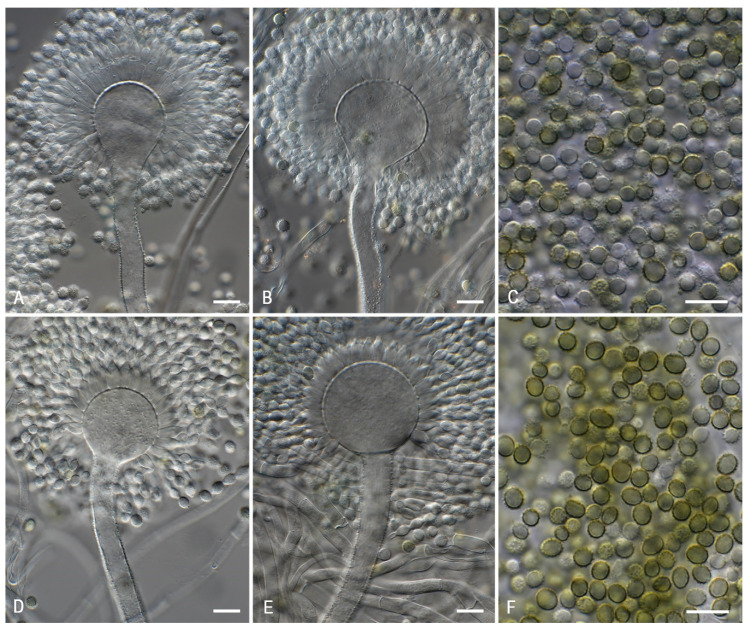
*Aspergillus novoparasiticus*. (**A**–**C**): CBS 108.30, (**D**–**F**): DTO 421-C3. (**A**,**B**,**D**,**E**). Conidiophores and conidia. (**C**,**F**). Conidia. Scale bar = 10 µm.

A polyphasic approach has become the gold standard in *Aspergillus* taxonomy. This approach is applied here for the identification and characterization of CBS 108.30, and the obtained data show that this strain is *A. novoparasiticus* and not *A. flavus,* as originally thought. Figure 1 shows the macromorphological features of CBS 108.30, as well as other *A. novoparasiticus* strains originating from different sources (soil, air, sputum of the leukemic patient, sugarcane, and sugarcane juice). As depicted in Figure 1, the majority of the investigated strains, including CBS 108.30, have a similar macromorphology: their growth rates on the tested agar media, sporulation patterns, and conidial colors are alike. Furthermore, the strains have similar growth rates on CYA incubated at 25, 37, and 42 °C. The exception is DTO 099-G4: this strain is slightly deviating in having darker brown conidia on CYA, MEA, and YES. *Aspergillus novoparasiticus* is originally described as being predominantly uniseriate, but biseriate structures could occur [11]. The microscopic examination of CBS 108.30 reveals predominantly biseriate conidiophores, and these structures are also observed in DTO 099-G4. In contrast, the recently isolated strains from Egyptian sugarcane juice are predominantly uniseriate (Figure 2).

**Figure 3:**
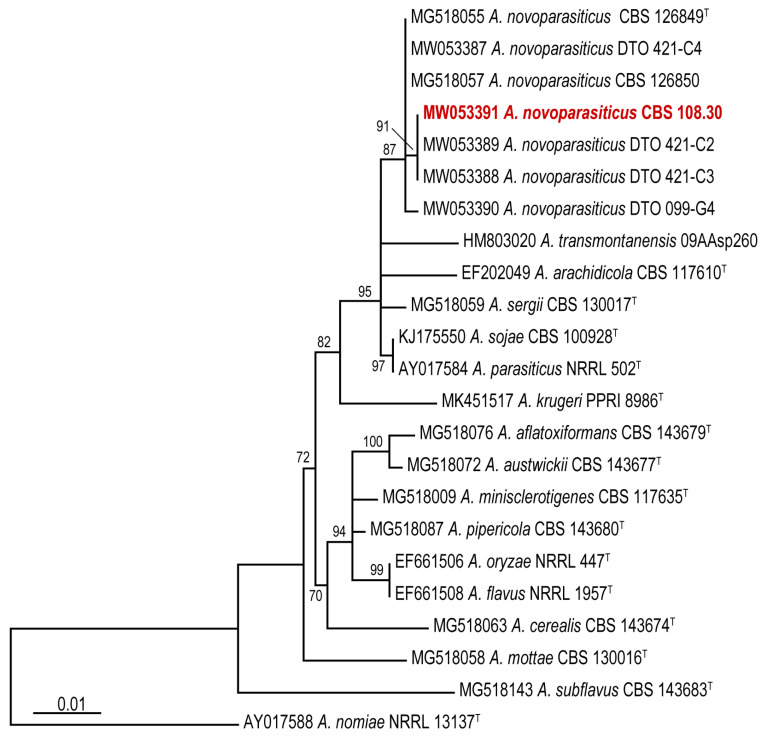
Phylogeny inferred from a *CaM* (calmodulin) nucleotide dataset using maximum likelihood (ML) analysis, showing the relationship of the species accommodated in *Aspergillus* section *Flavi* series *Flavi*. The bar indicates the number of substitutions per site. The bootstrap percentages of the ML analysis are presented at the node. Values less than 70% bootstrap support in the ML analysis are omitted.

Comparison of the generated *CaM* sequence of CBS 108.30 with other *A. novoparasiticus* strains and reference sequences of series *Flavi* species [12] confirms that this strain is *A. novoparasiticus*. CBS 108.30 resides in a clade together with other *A. novoparasiticus* strains are isolated previously from sugarcane juice in Egypt and from other sources in Brazil. High homology of 99.8% is observed with the partial *CaM* sequence of the ex-type strain *A. novoparasiticus* CBS 126849 (Figure 3).

Analysis of the exo-metabolites shows that a wide array of secondary metabolites, such as AFB_1_, AFG_1_, kojic acid, aspergillic acid, neoaspergillic acid, hydroxyaspergillic acid, neohydroxyaspergillic acid, leporin B, leporin C, flavacol, and *O*-methylsterigmatocystin, are produced by the investigated *A. novoparasiticus* strain (Table 1). Of those compounds, leporin B and C are reported for the first time for this species. These two metabolites (and/or other leporins) are also produced by *A. flavus* and *A. leporis* [13]. Although the toxicity of many secondary fungal metabolites in animals and humans are not confirmed, some of these toxic secondary metabolites might contribute to the overall toxicity, especially under chronic exposure scenario. However, some metabolites, such as kojic acid, are usually produced in enormous amounts by most species in *Aspergillus* section *Flavi* [5].

*Aspergillus novoparasiticus* was reported for the first time by Gonçalves et al. in 2012 as a new clinical species in Brazil [11]. It is now known that *A. novoparasiticus* is an aflatoxin type B and G producer [5]. Later, the species has been reported to occur in low frequencies in the cassava from Benin (two out of 20 identified isolates) [14], maize from Brazil (four out of 85 isolates) [15], traditional herbs called yerba mate from Brazil (three out of 60 isolates) [16], and maize from China (one out of 195 isolates) [17]. On the other hand, *A. novoparasiticus* is the predominant species in sugarcane and its by-products from Brazil (35 out of 57 isolated strains) [18] and Egypt (40 out of 44 isolates from sugarcane juice) [8]. Linking the current data with the results of Abdallah et al. [8], it can be concluded that sugarcane fields in Upper Egypt might be predominated with *A. novoparasiticus* for many decades. However, the predominance of this species needs a detailed investigation through a comprehensive survey study in Egyptian sugarcane fields, and such a study will reveal whether there is a certain association between *A. novoparasiticus* and sugarcane.

The old observation on the association between *A. novoparasiticus* (reported previously as *A. flavus*) and *Pseudococcus sacchari* on sugarcane is interesting, and it remains unknown whether this insect (or other insects) is involved in the spread and growth of *A. novoparasiticus* in Egyptian sugarcane, leading to aflatoxin contamination nowadays. All these data will be crucial to decide on the appropriate pre-harvest biocontrol strategy. For example, the application of non-aflatoxigenic *A. flavus* to control aflatoxin contamination in sugarcane might be ineffective because the actual contaminating species in the field is likely to be *A. novoparasiticus* [19]. The investigated *A. novoparasiticus* strains and other reports show that this species consistently produces aflatoxins. The occurrence of non-aflatoxin producers that can be used for biocontrol purposes should be aimed in a further study.

*Aspergillus novoparasiticus* has been included in a recent comparative genomics study of 23 *Aspergillus* section *Flavi* species to reassess their phylogenetic relationships [20]. This study with other previous survey studies indicates the increasing interest in exploring *A. novoparasiticus* as toxigenic species; however still, little is known on this species compared to other section *Flavi* species (e.g., *A. flavus* and *A. parasiticus*). Considering that sugarcane is an essential and strategic economic resource for several countries, it is absolutely necessary to pay more attention to this species as a possibly predominating mycotoxigenic species in sugarcane fields, and research is needed in order to reduce the potential crop loss and enhance the safety of sugarcane and its by-products.

Another critical issue that should not be ignored is the effect of climate change on *A. novoparasiticus*, *Pseudococcus sacchari,* and sugarcane crops. The impact of climate change on the ecology of mycotoxigenic fungi has recently gained considerable attention [21]. However, more research is still required to better understand the effect of climate change on toxigenic fungi in terms of pathogenicity and (multi) mycotoxin production. Taking into account the three parts (the fungus, the insect, and the crop) and their interaction under different scenarios of climate change will enable scientists to get more insight into the host–pathogen interaction and host resistance [22]. Besides, this will be pivotal to precisely evaluate the currently proposed prevention strategies against toxigenic fungi and mycotoxins i.e., whether they are going to be effective in the future or should be modified or replaced by other strategies. Moreover, studying the effect of climate change will assist in predicting any unexpected combination of mycotoxins or new emerging mycotoxins in the sugarcane field.

## Figures and Tables

**Table 1:** List of the extrolites produced by *A. novoparasiticus* CBS 108.30.

Metabolites	Chemical Formula	Exact Mass (Da)	Adduct (M+H^+^)	MS/MS Fragments (Da)	Retention Time (min)
AFB_1_	C17H12O6	312.06339	313.07066	298.0483, 285.0764, 241.0504, 214.0632	4.88
AFG_1_	C17H12O7	328.0583	329.06558	311.0562, 296.0342, 283.0612, 243.0661	4.58
Kojic acid	C6H6O4	142.02661	143.03389	125.0227, 97.0281, 69.0334	1.10
Aspergillic acid	C12H20N2O2	224.15248	225.15975	207.1507, 165.1024, 139.0491	4.35
Neoaspergillic acid	C12H20N2O2	224.15248	225.15975	207.1507, 165.1024, 123.0548	4.25
Hydroxyaspergillic acid	C12H20N2O3	240.14739	241.15467	312.1580, 181.1324, 153.0650, 100.0757	4.15
Neohydroxyaspergillic acid	C12H20N2O3	240.14739	241.15467	312.1580, 181.1324, 153.0650, 100.0757	3.99
Leporin B	C22H25NO3	351.18344	352.19072	278.1151, 216.0679,199.0608, 171.0654	8.08
Leporin C	C22H25NO2	335.18853	336.19581	254.1179, 214.0865, 200.0709, 188.0712	7.78
Flavacol	C12H20N2O	208.15756	209.16484	191.1529, 167.1185, 137.0704, 109.0743	5.40
O-methylsterigmatocystin	C19H14O6	338.07904	339.08631	324.0634, 306.0534, 295.0609, 295.0609	5.70
